# Assessment of Caregiver Burden and Burnout in Pediatric Palliative Care: A Path Toward Improving Children’s Well-Being

**DOI:** 10.3390/healthcare13131583

**Published:** 2025-07-02

**Authors:** Sefika Aldas, Murat Ersoy, Mehtap Durukan Tosun, Berfin Ozgokce Ozmen, Ali Tunc, Sanliay Sahin

**Affiliations:** Department of Pediatrics, University of Health Sciences, Mersin City Education and Research Hospital, 33800 Mersin, Turkey; ersoymurat33@yahoo.com (M.E.); mehtap.durukan@gmail.com (M.D.T.); dr.b.ozmen@hotmail.com (B.O.O.); alierentunc@hotmail.com (A.T.); sanliay@yahoo.com (S.S.)

**Keywords:** caregiving burden, caregiver burnout, pediatric palliative care, complex care needs, child health

## Abstract

Pediatric palliative care (PPC) is an evolving field that focuses on supporting children with life-limiting conditions, where the quality of care is vital. This study is a retrospective observational investigation that examines the experiences of caregivers to inform health and social service planning and enhance PPC quality. **Methods:** Data of pediatric patients aged 3 months to 18 years admitted to a PPC inpatient unit over two years were retrospectively reviewed. Sociodemographic characteristics of primary caregivers, including age, gender, number of siblings, education, income, occupation, and marital status, were recorded. Caregiver burden and burnout were assessed using the Zarit Burden Interview and the Maslach Burnout Inventory, respectively. Associations between caregiver characteristics and these measures were analyzed. **Results:** A total of 118 patients and caregivers were evaluated; 54.2% of patients were male. The most common diagnoses were neurological diseases (44.9%), followed by syndromic–genetic disorders (28.8%). About 34% of patients required more than three medical devices. Most caregivers were female (91.5%), mainly mothers and 53% had only primary education. No significant differences in care burden or burnout were found based on caregiver gender, marital status, or child’s diagnosis. However, the use of nasogastric tubes and multiple medical devices was associated with higher burnout. Lower income was significantly linked to higher care burden, while longer caregiving duration correlated with both increased burden and burnout. A moderate positive correlation was found between Zarit and Maslach scores. **Conclusions:** The complexity of PPC patients’ care increases caregiver burden and burnout. Expanding specialized PPC services is crucial to support caregivers and sustain home-based care.

## 1. Introduction

The global number of children requiring specialized health services has increased significantly due to the rising prevalence of chronic and life-limiting conditions. Advances in medicine, pharmacology, and bioengineering have improved survival for patients with congenital anomalies, prematurity complications, and neurological, metabolic, and oncological diseases [1]. Pediatric palliative care (PPC) takes a multidisciplinary approach, integrating medical, psychological, and rehabilitative support for children with complex health needs and their families, either in hospitals or at home. While palliative care was once viewed as end-of-life care, it is now widely accepted as essential from the point of diagnosis, continuing alongside curative therapies [2]. Although palliative care was traditionally associated with end-of-life care, it is now widely accepted that such services should commence at the time of diagnosis and continue alongside curative treatments [3]. According to the World Health Organization (WHO), around 20 million people require palliative care annually, of whom approximately 9% are children—indicating the global scale of pediatric need [4]. The WHO defines PPC as a comprehensive strategy aiming to relieve suffering among children with life-threatening illnesses and their families across physical, emotional, social, and spiritual domains [5]. The American Academy of Pediatrics (AAP) similarly advocates for the integration of palliative care within healthcare systems, emphasizing its importance regardless of whether a child’s condition is curable or ultimately fatal [6]. PPC is recognized as a holistic approach that neither hastens nor postpones death but rather focuses on improving the quality of life for children with life-limiting conditions. This model emphasizes comprehensive support not only for the child but also for their family throughout the continuum of care. However, this complex care process introduces substantial challenges for family caregivers, who frequently bear intense emotional, physical, and financial burdens. Over time, these cumulative stressors can negatively affect caregivers’ psychological and physical well-being. Research has linked caregiving in such contexts to increased rates of psychiatric disorders, particularly anxiety, depression, and clinical burnout syndrome, underscoring the psychological vulnerability of this population [1,7,8]. There is a well-documented interplay between caregiving burden and caregiver burnout, particularly in the context of pediatric palliative care. As the intensity and complexity of caregiving responsibilities increase—such as managing advanced medical technologies, administering multiple medications, or providing long-term care for children with chronic conditions—the risk of emotional exhaustion, depersonalization, and diminished personal accomplishment rises markedly. These domains, which constitute the core dimensions of burnout, are frequently exacerbated in caregivers operating under sustained stress [8,9].

This study aimed to evaluate the caregiving burden and burnout levels among primary caregivers of children hospitalized in a tertiary pediatric palliative care unit. It also sought to examine how caregivers’ sociodemographic characteristics, caregiving context, and clinical factors related to burden and burnout. By identifying these associations, the study contributes empirical data that can inform future care models and targeted caregiver support interventions.

## 2. Materials and Methods

### 2.1. Study Design and Setting

This retrospective observational study was conducted at the Pediatric Palliative Care Unit of a tertiary Children’s Research Hospital. The unit includes 24 inpatient beds, all of which are specifically designated for pediatric palliative care. The study period covered admissions from January 2024 to March 2025. The research was approved by the Ethics Committee of Toros University (approval number: DEISC-PR-25.09.2024/157) and was carried out by the ethical standards of the Declaration of Helsinki. Although no formal power analysis was performed, the sample size aligns with previous cross-sectional studies in pediatric palliative care, which typically include 80 to 100 participants [10].

### 2.2. Participants

The study included pediatric patients aged between 3 months and 18 years who were hospitalized in the PPC unit, along with their primary caregivers. Inclusion criteria for caregivers were as follows: age ≥18 years, cohabiting with the child, and being directly responsible for the child’s care for at least three months. Interviews were conducted by trained nursing staff who were familiar with the clinical environment but were not directly involved in the care planning or medical management of the patients. To minimize potential interviewer bias, all assessments were conducted using a standardized, structured protocol.

### 2.3. Variables and Data Collection

In this study, clinical data regarding the patient’s medical history were retrospectively obtained from hospital records. However, the assessment of caregiver burden and burnout was conducted through a face-to-face, observational approach during the hospitalization period, using structured interviews with primary caregivers. This hybrid data collection method allowed for the integration of retrospective clinical information with prospectively gathered psychosocial data. No missing data were observed in the analyzed data. For pediatric patients, the following variables were recorded:

- Age;
- Gender;
- Primary diagnosis;
- Disease duration;
- Number and type of medical devices used (e.g., mechanical ventilation, tracheostomy, PEG, NGT);
- Number of medications;
- Number of siblings.

Caregivers’ sociodemographic characteristics were also recorded, including:

- Age;
- Gender;
- Relationship to the child;
- Educational background;
- Monthly income level;
- Occupation;
- Marital status;
- Data collection was conducted through structured face-to-face interviews by trained personnel. Each session lasted approximately 20–30 min. During the interview, participants completed a structured questionnaire, the Zarit Burden Interview (ZBI), and the Maslach Burnout Inventory (MBI).

### 2.4. Assessment Tools

#### 2.4.1. Zarit Burden Interview (ZBI)

The ZBI is a 22-item scale that evaluates caregivers’ perceived burden. Each item is scored on a 5-point Likert scale from 0 (“never”) to 4 (“almost always”). Total scores range from 0 to 88, with higher scores indicating greater burden. The scale assesses multiple domains, including physical, emotional, financial, and social aspects of caregiving [11].

#### 2.4.2. Maslach Burnout Inventory (MBI)

The MBI consists of 22 items evaluating three dimensions of burnout: Emotional Exhaustion, Depersonalization, and Reduced Personal Accomplishment. Items are rated on a 5-point Likert scale from 1 (“never”) to 5 (“every day”). Higher scores on the first two subscales and lower scores on the third indicate higher burnout. Participants scoring ≤46 were classified as having low burnout, and those scoring >47 as having high burnout The cut-off scores used in this study were based on the original classification by Maslach and Jackson [12,13].

Cronbach’s alpha coefficients were 0.82 for the ZBI and 0.74 for the MBI, demonstrating acceptable internal consistency.

### 2.5. Statistical Analysis

Data were evaluated using the IBM SPSS 26 (Statistical Package for Social Sciences) program. Descriptive statistics were determined as a number, percentage (%), ratio, median, interquartile range (IQR = Inter Quantile Range, minimum, and maximum). Normality was evaluated by the Shapiro–Wilk test. For descriptive statistics, categorical variables were presented as numbers and percentages, and continuous variables were presented as median (minimum–maximum value). When data did not follow a normal distribution, the Mann–Whitney U test was used to compare two independent groups. The Kruskal–Wallis test was used to compare three or more independent groups. Spearman’s Rho Correlation Analysis was used for the correlation of continuous variables with non-normal distribution. If the absolute value of the correlation coefficient was 0.00, there was no correlation; 0.01 to 0.25 was weak; 0.26 to 0.50 was moderate; a correlation between 0.51 and 0.75 was considered a strong correlation, and a correlation greater than 0.75 was considered a very strong correlation. This classification was based on the interpretation criteria described by Dancey and Reidy [14]. Statistical significance was determined at a *p* < 0.05 level.

## 3. Results

In our study, a total of 118 patients hospitalized in the PPC unit and their caregivers were evaluated. Of the patients included in the study, 64 (54.2%) were male, and 54 (45.8%) were female. Diseases of patients included neurological disease (cerebral palsy, epilepsy, neurometabolic diseases, etc.) (44.9%), syndromic–genetic diseases (28.8%), HİE sequelae, hypotonic infant (9.3%), post-traumatic injuries (accident, drowning, etc.) (8.5%), neuromuscular diseases (5.1%), and meningomyelocele (3.4%), respectively. Sixty-seven patients (56.7%) required care for more than 2 years. While 93 (78.8%) of the patients used less than five medications, 25 (21.2%) needed five or more medications. Demographic data such as age, gender, disease duration, diagnosis, medical devices used, and number of siblings are shown in Table 1.

When the data of caregivers were analyzed, it was found that 108 (91.5%) were female and 10 (8.5%) were male. In this group, the majority, 99 (83.9%), were women and mothers of sick children, 12 (10.2%) were fathers, and 7 (5.9%) were relatives or other nursing staff. Demographic data of the caregivers are given in Table 2.

When the care burden and burnout scores were compared, no statistically significant difference was observed regarding gender (*p* > 0.05). The care burden felt by the caregivers was determined as light, at 39.8%. Maslach burnout scores in patients with NG were statistically significantly higher (*p* < 0.05), with a median of 56 (IQR: 48–57) compared to those without NG, with a median of 53 (IQR: 50.5–61.5).

Although no statistically significant correlation was found between the number of medical devices children had to use to survive and the care burden perceived by the caregivers (*p* > 0.05), a significant correlation was observed with the total Maslach burnout score (*p* < 0.05). The evaluation of the Maslach burnout and Zarit care burden scores of caregivers according to the patients’ medical device dependency is given in Table 3.

No statistically significant associations were identified between the child’s diagnosis, caregiver’s gender, or the marital status of the child’s illness and the levels of perceived care burden and burnout (*p* > 0.05). Similarly, the monthly income level of caregivers was not significantly associated with total Maslach Burnout Inventory (MBI) scores (*p* > 0.05). However, a statistically significant relationship was observed between income and perceived care burden (*p* < 0.05). Furthermore, analysis of the relationship between the duration of caregiving and both burnout and perceived care burden revealed a statistically significant association with the duration of the child’s illness (*p* < 0.05). Although the number of siblings was included as a variable in the study, no statistically significant association was found between this factor and caregiver burden or burnout scores (*p* > 0.05). Comparison of Zarit burden scale and Maslach burnout scale is given in Table 4.

There was a moderate positive correlation between Maslach and Zarit scores with r = 0.294, and this relationship was statistically significant (*p* = 0.001).

## 4. Discussion

Pediatric palliative care (PPC) is a developing field, yet studies addressing caregiver burden remain limited. Due to scarce pediatric-specific data, comparisons with adult palliative care are often necessary. In our study, neurological conditions were the most frequent diagnostic group, consistent with prior research [15]. Similarly, a 2024 study in Portugal reported a prevalence of neuromuscular disorders at 41.2 per 100,000 under age 18, mostly with genetic origins [16]. Genetic disorders ranked second in our cohort, likely reflecting high rates of consanguineous marriage and limited access to genetic counseling.

Polypharmacy was common, with over half of patients prescribed five or more daily medications—a practice strongly linked to increased caregiver burden [17,18]. Our findings align with this and emphasize the dual impact of treatment complexity on both patients and caregivers. Over half of the patients had been receiving care for more than two years. Longer illness duration correlated with higher caregiver burden but not with burnout—echoing similar studies [19].

The use of nasogastric (NG) tubes was significantly associated with increased burnout scores in caregivers. Only one prior study has directly examined this link [13]. Nasogastric (NG) tubes may contribute to caregiver burnout due to the high frequency of maintenance, the need for continuous monitoring, and the emotional stress associated with feeding difficulties and the risk of aspiration. Unlike other medical devices, NG tubes demand direct and repeated involvement from caregivers in daily care routines. Moreover, improper placement or accidental dislodgement may pose immediate clinical risks, leading to heightened caregiver anxiety. These factors increase emotional exhaustion, contributing to higher burnout scores. Prior studies have similarly reported elevated stress among caregivers managing NG tube feeding at home, citing both technical complexity and emotional burden during post-discharge transitions [20,21]. Our findings were consistent with the results which indicate that NG tube use is associated with increased caregiver burnout compared to other medical interventions.

Caring for children using three or more medical devices appears to increase caregiver burnout. Procedures such as secretion aspiration and home ventilator monitoring in tracheostomized patients impose high costs and significant responsibility. The maintenance of devices like tracheostomy cannulas and ventilator circuits, along with the provision of feeding materials, creates both psychological and financial strain [22,23]. For children requiring NG or PEG feeding every 3–4 h, caregivers spend 3–4 h daily on feeding and treatments, limiting time for personal needs and complicating time management.

In this study, the presence and number of siblings in the family did not have a significant effect on the care burden and burnout level perceived by the caregiver, in line with the literature [13].

In this study, 91.5% of caregivers of PPC patients were women, and notably, 83.9% were the mothers. This was consistent with studies which have reported a higher percentage of female caregivers for patients requiring palliative care [13,24,25]. A recent study examining gender differences in caregiving for children with genetic or rare diseases found that women more often assumed the role of primary caregiver and experienced higher levels of parental stress and depressive symptoms compared to men. These findings suggest that mothers tend to perceive their child’s illness more difficult to manage, contributing to a greater emotional burden [26]. In our study, it was also difficult to assess the effect of gender on burnout levels because the number of male participants was quite low. A study in 2024 found that female caregivers providing home palliative care to advanced cancer patients experienced more psychological difficulties and felt more significant psychological support needs [27]. One study indicated that men perceive a greater burden of care than women [28]. However, our study did not find a significant difference between male and female caregivers in terms of perceived care burden or burnout levels.

Although most caregivers in our study had only basic education, no significant relationship was found between educational level and perceived burden or burnout. This suggests that caregiver stress may be influenced more by personal, psychosocial, and environmental factors than education alone.

The finding that 61.1% of caregivers reported earnings below the national minimum wage underscores a critical socioeconomic vulnerability in this population. Caregivers experience significant financial pressures, including direct out-of-pocket expenses, opportunity costs, and lost employment income [29]. These financial burdens are linked to increased psychological distress, as noted in pediatric settings where hospital admission exacerbates acute and chronic financial strain [30]. An U.S.-based multicenter study found a financial hardship among parents of children receiving PPC, highlighting the burden low-income places on caregivers [31]. Given that inadequate financial resources may compound caregiver burnout and jeopardize home-based palliative care, targeted policy interventions such as caregiver stipends, paid leave, and easier access to social services are urgently needed. Without such support, families are at risk of experiencing compounded social and psychological hardship, which could in turn undermine their ability to provide consistent, quality of care. According to WHO, 98% of individuals needing palliative care live in low- and middle-income countries, nearly half being in Africa [2]. Since 2022, our country, Turkey, has been classified within this category. Factors such as migration, refugee influx, health tourism, and regional conflicts increase PPC needs. In our study, many families reported difficulty affording essential medical devices due to inadequate government support and rising costs linked to exchange rate fluctuations, contributing to caregiver burden and burnout [32]. In our study, it was found that burnout increases as care burden increases. Similar findings were reported by Egici et al., who used the same scales in adult populations, and it was reported that an increase in care burden was associated with higher burnout levels [33]. Unsar et al. reported that, anxiety and depression were more pronounced in caregivers of advanced cancer patients. These findings emphasize the importance of psychological support for caregivers and suggest that healthcare professionals should consider the factors that increase the care burden [34]. Interviews revealed that families, particularly those of SMA patients, required greater psychological and financial support. The burden of securing funding for the high-cost gene therapy Zolgensma, which is covered by insurance in many European countries but not in our country, causes significant stress and hinder their ability to focus on caregiving. A study by Dogan et al. assessed the caregiving burden among individuals caring for children with chronic illnesses using the Zarit Scale, revealing that 29.4% experienced a moderate level of burden, while 7.5% reported a high level [35]. These results were consistent with the findings of our study. A latest study examining burnout among PPC providers found that, while personal growth and religious beliefs play a mitigating role, elevated caregiving demands were significantly associated with increased levels of burnout [36]. In another adult study conducted [37] on caregivers of Alzheimer’s patients, it was found that burnout increased as the care burden increased, consistent with those observed in our study.

To the best of our knowledge, our study focuses on pediatric patients in need of palliative care and their caregivers, filling an important gap in this field. Our results were closely in line with studies on adults in various disease groups. While it is acknowledged that PPC differs significantly from adult palliative care, we observed similar effects on caregiver groups in both contexts. However, this area of research is under-explored, and additional studies are required, since the distribution of diseases and care strategies differs from that of adults.

## 5. Conclusions

PPC patients require prolonged and technologically complex support, placing substantial emotional and physical demands on primary caregivers. Increased caregiving burden correlates with higher burnout levels, potentially compromising quality of care. To mitigate this, healthcare systems should implement routine screening for caregiver burnout, integrate structured psychosocial support programs, and ensure financial assistance for families managing long-term, device-dependent care. Additionally, expanding multidisciplinary PPC centers with caregiver-focused services could enhance the continuity of care and outcomes. These findings may improve policy development and resource allocation in countries with similar healthcare and socioeconomic challenges.

## 6. Limitations

The limitation of this study is that the assessment of burnout was based on cross-sectional data collected in a single center design. As such, it may not fully capture the daily or situational fluctuations in stress and emotional strain that are particularly common among pediatric caregivers. Given the unpredictable and intensive nature of caregiving tasks, especially in cases involving medical devices or feeding complications, burnout levels may vary considerably within short timeframes. Future studies employing longitudinal or ecological momentary assessment methods could provide a more nuanced understanding of these dynamic experiences.

## Figures and Tables

**Table 1:** Demographic data of the patients, diagnosis distribution, and the medical devices used by patients in need of palliative care.

Variables (n = 118) (%)	
Gender, n (%)	
Female	54 (45.8)
Male	64 (54.2)
Age (months), Median (min-max)	59 (4–214)
Duration of Disease (months), Median (min–max)	36 (3–210)
Diagnosis, n (%)	
Neurological Disease (Cerebral Palsy, Epilepsy, Neurometabolic Diseases, etc.)	53 (44.9)
Syndromic-Genetic Diseases	34 (28.8)
HİE Sequelae/Hypotonic Infant	11 (9.3)
Post Traumatic Injury (Accidents, Drownings, etc.)	10 (8.5)
Neuromuscular Diseases	6 (5.1)
Meningomyelocele	4 (3.4)
Medical technology, n (%)	
Nasogastric tube (NG)	29 (24.6)
Percutaneous Entero-Gastrostomy (PEG)	58 (49.2)
Tracheostomy	29 (24.6)
Non-Invasive Ventilator (BIPAP)	10 (8.5)
Home Ventilator Dependent	20 (16.9)
Central Venous Catheter (CVC)	74 (62.7)
Ventriculo-Peritoneal Shunt (VP Shunt)	13 (11.0)
Wheelchair-Dependent	30 (25.4)
Number of medical technologies used, n (%)	
One Medical Device	21 (23.7)
Two Medical Devices	50 (42.4)
3> Medical Devices	40 (33.9)
The patient’s brother/sister	
Yes	93 (78.8)
No	25 (21.2)

**Table 2:** Demographic data of caregivers.

Variables (n = 118) n (%)	
Age (years), Median (min-max)	35.5 (21–65)
Gender	
Female	108 (91.5)
Male	10 (8.5)
The degree of the relationship	
Mother	99 (83.9)
Father	12 (10.2)
Others	7 (5.9)
Educational Status	
No education	11 (9.3)
Primary education	62 (52.6)
High school	35 (29.7)
University	10 (8.4)
Marital status	
Married	109 (92.4)
Never married (single parent)	3 (2.5)
Widow	3 (2.5)
Divorced/Living Separated	3 (2.5)
Income rate	
<20,000 ₺ (Below Minimum Wage)	72 (61.1)
20,000–30,000 ₺	23 (19.5)
30,000–60,000 ₺	21 (17.8)
>60,000 ₺	2 (1.7)
Working	
Yes	13 (11.0)
No	105 (89.0)
Job	
Housewife	104 (88.1)
Officer	4 (3.4)
Employee	1 (0.8)
Self-employment	2 (1.7)
Other	7 (5.9)
Would the caregiver want to work if they had the opportunity?	
Yes	67 (56.8)
No	51 (43.2)
Would the caregiver like to be supported?	
Financial aid	39 (33.1)
Get a job or work	12 (10.2)
Both	67 (56.8)

**Table 3:** Evaluation of Maslach burnout and Zarit care burden scores of caregivers according to patients’ medical device dependency.

Medical Technology Used	Maslach Burnout Inventory Median (Q1–Q3)	*p*-Value	Zarit Burden Interview Median (Q1–Q3)	*p*-Value
Any technology (≥1 device)	56 (50.0–61.0)	–	30 (25.0–35.0)	–
NG	53 (48.0–57.0)	<0.05	29 (25.5–33.5)	0.623
PEG	58.5 (50.75–61.25)	>0.05	30 (24.75–35.0)	0.625
Tracheostomy	58 (52.0–62.0)	>0.05	28 (25.0–35.0)	0.481
NIV	56 (50.0–61.0)	>0.05	34.5 (25.5–36.0)	0.376
MV Dependent	53.5 (46.75–56.7)	>0.05	27.5 (23.5–34.0)	0.197
CVC	55 (48.0–60.0)	>0.05	29 (24.0–35.0)	0.351
VP Shunt	54 (45.0–61.0)	>0.05	32 (23.5–37.5)	0.591
Wheelchair Use	57 (47.75–60.25)	>0.05	31 (21.0–36.75)	0.699
≥3 Technological Devices Used	56 (48.25–60.0)	<0.05	28.5 (25.0–35.0)	0.432

Mann–Whitney U, NG: nasogastric feeding tube, PEG: Percutaneous Enteral Gastrostomy, NIV: Non-invasive ventilator, CVC: Central Venous Catheter, VP Shunt: Ventriculoperitoneal Shunt.

**Table 4:** Comparison of Zarit burden scale and Maslach burnout scale.

	Maslach		Zarit	
	r	*p*	r	*p*
Caregiver Age	0.181	0.049	0.044	0.409
Education level	0.064	0.492	0.042	0.633
Number of siblings	0.141	0.127	0.026	0.78
Monthly Income	0.143	0.122	0.173	0.027
Duration of the Disease	0.067	0.469	−0.059	0.042
Child Age	0.179	0.052	−0.015	0.869
Number of technological devices	−0.038	0.68	−0.123	0.183
Number of Drugs Used > 5	0.053	0.57	0.05	0.043

Spearman’s rho Test, r: Correlation coefficient.

## Data Availability

The data supporting the findings of this study are available from the database of Mersin City Training and Research Hospital upon reasonable request.
